# Mental health impact of covid on athletes

**DOI:** 10.1192/j.eurpsy.2021.1067

**Published:** 2021-08-13

**Authors:** K. Shah, S. Jain, I. Glick

**Affiliations:** 1 Department Of Psychiatry, Griffin Memorial Hospital, Norman, United States of America; 2 Psychiatry, Texas Tech University Health Sciences Center, Midland, United States of America; 3 Psychiatry, Stanford University School of Medicine, Stanford, United States of America

**Keywords:** Covid, Mental Health of Athletes, sports psychiatry

## Abstract

**Introduction:**

The coronavirus pandemic continues to impact all aspects of the daily life of the public worldwide. With decreased economic activity, the sports industry faces significant challenges of maintaining athletes’ mental health while seeking the best strategies for eventual return to sports competition.

**Objectives:**

We aim to evaluate COVID-19 related factors impacting on the mental health of athletes and provide appropriate management steps.

**Methods:**

We examined MeSH terms “Athletes,” “Sports,” “COVID-19,” in the context of “Mental Health,” “Mental Disorders,” “Behavioral Medicine,” “Risk Factors.” We identified seven studies for the qualitative synthesis per the PRISMA guidelines, searching Medline, PubMed, PubMed Central, and PsychInfo databases until July 2020.

**Results:**

The pandemic has negatively impacted athletes’ mental wellbeing due to decreased physical activities, limited resources, fears, and delays or cancellations of the sporting event. The negative psychological impact on athletes is due to self-isolation measures leading to worries of less preparedness for the lockdown, reduced physical activity, loss of competitive advantages, fear of being infected, social isolation, and loneliness. During this period, athletes struggled to maintain baseline routine and engaged in excessive calorie intake, eating low-quality food, substance use, and sleep disruption. It has caused anxiety, depression, PTSD, and mood disorder at varying degrees of severity in athletes.

**Conclusions:**

Limited resources during a pandemic have caused adverse mental impact on athletes. We recommend improving physical activity through confined or virtual training programs with colleagues. A collaborative approach is required by clinicians, psychologists, coaches, sports organizations, government bodies to limit the pandemic’s mental health impact.

